# Highly Efficient Single-Layer Phosphorescent Organic Light-Emitting Diodes Based on Co-Host Structure

**DOI:** 10.3390/ma13092029

**Published:** 2020-04-26

**Authors:** Tianyu Zhang, Asu Li, Ren Sheng, Mingyang Sun, Ping Chen

**Affiliations:** 1 Key Laboratory of Geophysical Exploration Equipment, Ministry of Education, College of Instrumentation and Electrical Engineering, Jilin University, Changchun 130000, China; zty@jlu.edu.cn (T.Z.); lias19@mails.jlu.edu.cn (A.L.); smy19@mails.jlu.edu.cn (M.S.); 2 State Key Laboratory of Integrated Optoelectronics, College of Electronic Science and Engineering, Jilin University, Changchun 130012, China; poiuytr_000@sian.com

**Keywords:** single-layer OLEDs, uniform co-host structure, exciton recombination region, dopant effect, charge carriers

## Abstract

High-efficiency single-layer organic light-emitting diodes (OLEDs) based on a simple structure doped with iridium(III) bis(4-phenylthieno[3,2-c]pyridinato-N,C2′) acetylacetonate (PO-01) as emission dyes are realized, achieving maximum current efficiency (CE) and power efficiency (PE) of 37.1 cd A^−1^ and 33.3 lm W^−1^ as well as low turn-on voltage of 3.31 V. Such superior performance is mainly attributed to the employment of a uniform co-host structure and assisted charge transport property of phosphors dyes, which were in favor of the balance of charge carrier injection and transport in the single emitting layer (EML). Moreover, systematic researches on the position of exciton recombination region and the dopant effect on charge carriers were subsequently performed to better understand the operational mechanism. It could be experimentally found that the orange emitting dopants promoted the acceleration of the charge carriers transport and raised the exciton recombination efficiency, eventually leading to an excellent performance of single-layer OLEDs.

## 1. Introduction

Organic light-emitting diodes (OLEDs) have gained considerable focus on the applications in both lighting technology and organic flat-panel display due to their high efficiencies, self-emission properties, and compatibility with ease of processing and reduced power consumption [1,2,3,4,5].

Phosphorescent OLEDs (PhOLEDs) represent an effective way to obtain advanced performance due to their intrinsic property of harvesting both singlet and triplet excitons, attaining maximum internal quantum efficiency of nearly 100% [6,7,8,9,10]. Generally speaking, in order to realize excellent performance, one universal strategy is the adoption of the multilayer structures which include carriers transport layers, exciton blocking layers, or extra space-layers to eliminate exciton quenching and balance charge carriers [11]. However, fabricating OLEDs with multiple layers will inevitably increase the use of materials and fabrication steps, presenting new obstacles to the commercial application of OLEDs. In consequence, how to simplify the device structure and balance the charge carriers seem to be very meaningful and imperative. Note that the single-layer OLED with only one organic layer is considered as a promising approach for its simplified structure [12].

Recently, single-layer OLEDs have aroused extensive interests due to their simplest structures and superior performance [13,14]. A variety of methods have been suggested to simplify the device structure, among which adopting a bipolar organic material is an efficient one [15,16,17,18]. Hsu et al. demonstrated a new bipolar host material for the single-layer blue phosphorescent device employing single-layer structure, which achieved peak CE of 35.4 cd A^−1^ [19]. Chang et al. reported efficient single-layer blue PhOLEDs based on a novel bipolar host material, attaining maximum CE of 21.3 cd A^−1^ [20]. Excellent performance has been achieved by using these bipolar materials, however, direct research on the synthesis of such host materials has been rather restricted and single-layer OLEDs optimizing the bipolar materials are still rare. Based on these facts, it is thus urgently required to develop new device concept for single-layer devices with enhanced performance and simplified structure.

In this study, we presented single-layer devices with superior performance based on uniform co-host structure. The fabricated OLEDs employed the PO-01 as dopant, acquiring maximum CE and PE of 37.1 cd A^−1^ and 33.3 lm W^−1^, respectively, and low turn-on voltage. The improvements were mainly derived from the adoption of co-host structure, which was beneficial for achieving balanced charge transport. Additionally, the operational mechanism had been analyzed in the field of exciton recombination region and the effect of dopant molecules on charge carriers transport. It could be concluded that the outstanding bipolar property of the co-host materials helped to balance the charge transport and broaden the recombination region, leading to efficient performance.

## 2. Materials and Methods

**Figure 1:**
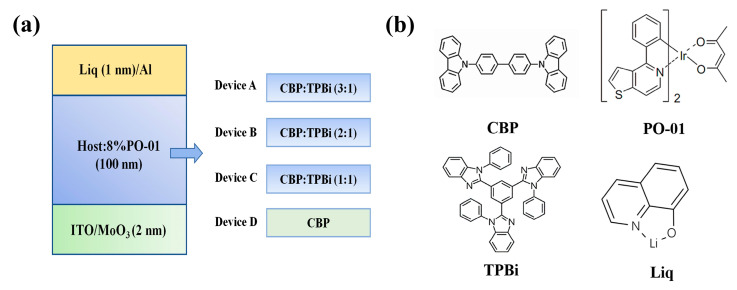
(**a**) The structure of devices A–D; (**b**) the chemical structures of the used materials.

Figure 1a detailed the structure of devices A–D. The resulting devices were fabricated on ITO-coated glass substrates, which were pre-cleaned in an ultrasonic bath with acetone, ethanol and deionized water in succession, followed by dried at 120 °C and ultraviolet (UV) ozone treatment prior to film deposition. Here, ITO covered by 2 nm MoO_3_ was served as composite anode. Then, 4,4′-bis(N-carbazolyl)biphenyl (CBP) with high hole mobility and 1,3,5-tris(2-N-phenylbenzimidazolyl) benzene (TPBi) with high electron mobility were blended as the co-host to construct an appropriate device configuration. PO-01 uniformly doped in the four devices functioned as the orange phosphorescent emitter. Both 1 nm Liq and 100 nm Al with an active area of 3 mm × 3 mm were used as composite cathodes. Consequently, ITO/MoO_3_ and Liq/Al were used as composite anode and composite cathode, respectively. Furthermore, only one organic EML was used to realize the light emitting and the charge transport. The devices A–D were only made of electrodes and one EML according with the characteristics of single-layer configuration [21]. Further, the test of device performance was a very crucial step in data analysis. The electrical property of the fabricated OLEDs was investigated through a Keithley 2400 source-meter unit linked with a PR655 spec-scan spectrometer (Ruixuan Electronic Technology (Shanghai) Co. Ltd., Shanghai, China) simultaneously.

## 3. Results and Discussion

To investigate the effect of using uniform co-host composition on the devices performance and address the issue of charge balance, we prepare devices employing the following structure: ITO/MoO_3_ (2 nm)/host: 8% PO-01 (100 nm)/Liq (1 nm)/Al, where the host can be either a mixture with the doping ratio CBP to TPBi assigned by 3:1, 2:1, 1:1, and pure CBP host as a comparison, corresponding to devices A, B, C, and D. PO-01 with the doping concentration of 10 wt.% was kept throughout the emitting layer.

**Figure 2:**
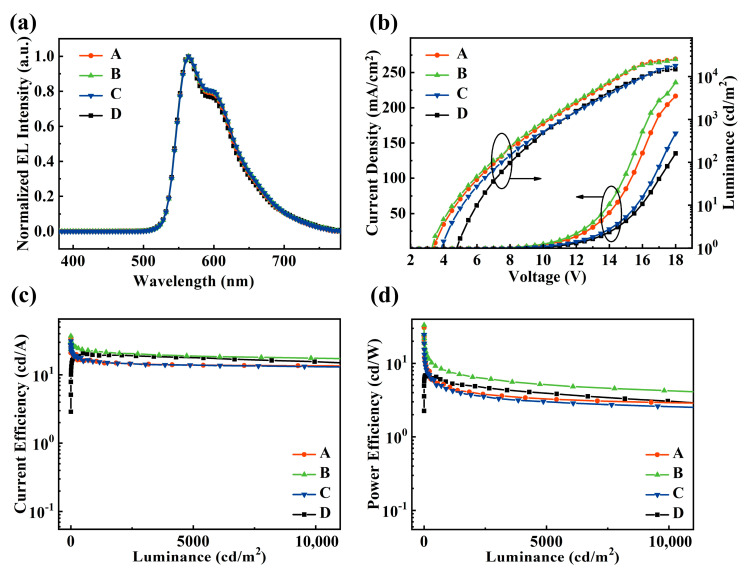
(**a**) The normalized electroluminescence (EL) spectra of devices A–D at 9 V; (**b**) the current density-voltage-luminance, (**c**) the CE-luminance, (**d**) the PE-luminance curves of devices A–D.

To simplify discussion, all detail performance date is summarized in Table 1. As shown in Figure 2a, the emission peak can be clearly observed at around 650 nm, which originates from the PO-01 dopant. Moreover, a single emission peak without any other sub-peaks from shorter and longer wavelength indicates the efficient energy transfer from host, suggesting the generated excitons are excellent confined on PO-01. As shown in Figure 2b, the current densities of devices A–C using co-host structure are much higher as compared with device D. For instance, the current densities of devices A–D is 135.8, 166.4, 73.2, 63.7 mA cm^−2^ at 16 V, respectively. The improvement should be ascribed to that the introduction of TPBi with high electron-transport property in the co-host system facilitates the transport of electrons compared to that with pure CBP host [22]. Furthermore, the turn-on voltage (V_on_) is 3.31 V in device B, which is considerably lower than that of device D of 4.81 V on account of the reduced energy barriers between the electrode and host materials. Both of the low brightness and high turn-on voltages in device D imply that the CBP-hosted device performs with poor charge balance. In addition, the enhancement in brightness of device B also implies an improvement of charge balance over device D.

For device D with pure CBP host, the charge transport balance is poor because the hole mobility of CBP is about twice faster compared to electron, which leads to lower performance. With the introduction of TPBi, the mobility of charge carriers in Device A (CBP:TPBi = 3:1) tends to be balanced compared to device D, leading to increasing efficiency. Then, with increasing doping ratio of TPBi, the CE of device B (CBP:TPBi = 2:1) shows increasing tendency for superior charge balance, and then shows a considerable reduction when the doping ratio varies from 2:1 to 1:1, which infers that the excessive concentration of TPBi in device C (CBP: TPBi = 1:1) leads to low device performance due to imbalanced charge carriers transport. Moreover, the highest efficiency is achieved by device B with maximum CE of 37.1 cd A^−1^ and the CE keeps as high as 22.1 cd A^−1^ even when the luminance increases to 1000 cd m^−2^, whereas the device D achieves peak efficiency of 20.7 cd A^−1^. In summing up the results above, we conclude that suitable introduction of electron transport-type material (TPBi) into CBP host results in highly improved efficiency, as well as reduced driving voltage and balanced carriers transport.

As we know, due to the absence of additional carriers or excitons blocking layers in the single-layer devices, the charge recombination efficiency shows a direct ratio on the balance of holes and electrons. The imbalance of charge injection and transport inevitably results in unsatisfied device performance and exciton quenching effect owing to the shift of the recombination region toward to electrodes [12,23]. Thus, it is an essential way to avoid the negative effects by keeping the recombination region away from electrodes. Further, the position of recombination region is an important factor affecting the device performance. Moreover, in order to clarify the location of the main exciton recombination region in the EML of device B, we prepare another serious of multilayer devices P_1_–P_4_ where an ultrathin orange sensing layer of 0.15 nm is inserted at x nm away from the composite anode. The structure of the four devices based on device B with the doping concentration CBP to TPBi modulated by 2:1 here is ITO/MoO_3_ (2 nm)/co-host (x nm)/PO-01 (0.15 nm)/co-host [(100-x) nm]/Liq (1 nm)/Al, where the x is set to be 40, 50, 60, 80 for devices P_1_–P_4_, respectively.

**Figure 3:**
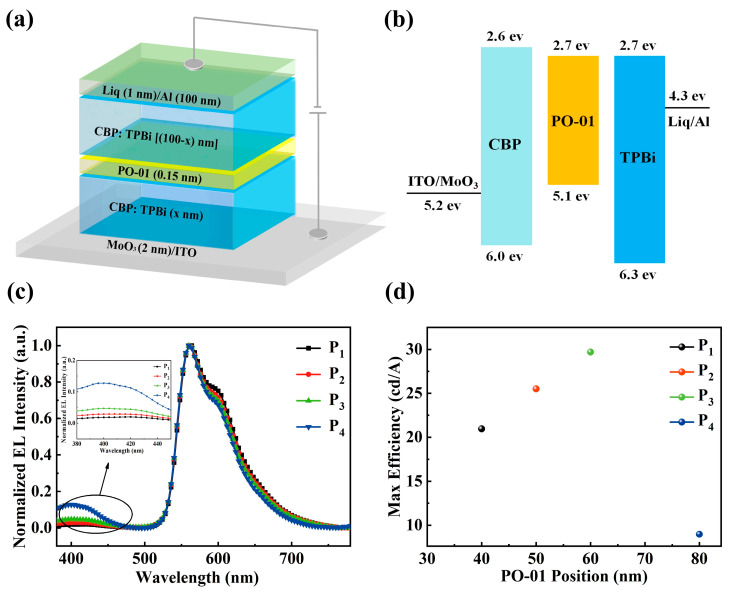
(**a**) The structure of devices P_1_–P_4_; (**b**) the specific energy level diagram of the used materials; (**c**) the normalized EL spectra of devices P_1_–P_4_ at 7 V (inset: partial magnified EL spectra); (**d**) the maximum CE-PO-01 position characteristics of devices P_1_–P_4_.

As shown in Figure 3, the spectra show one main emission peaks originating from PO-01 in devices P_1_–P_3_. Moreover, upon moving the ultrathin layer toward the cathode side, the emitting intensity of host materials at short wavelength gradually increased, resulting from the insufficient energy transfer from host to PO-01. In Figure 3d, device P_4_ performs with poor performance revealing that the energy transfer efficiency is low from host to dopant and the main exciton recombination region is far away from cathode. The CE increases gradually with x varying from 40 to 60 until the strongest efficiency of 29.7 cd A^−1^ is achieved in device P_3_, fully indicating that the exciton recombination region should be located in the central region of the EML for balanced transport of charge carriers. As is previously stated, this kind of exciton distribution greatly declines the exciton quenching effect in transport layer, which should be one of the major reasons for the excellent performance of device B. Thus, we conclude the appropriate composition ratio of the co-host materials is favorable for exciton distribution and hence balanced charge carriers [24].

The dopant molecules play critical part in charge carriers transport as mentioned in previous report, which would have greatly impact on carrier mobility and device performance especially in single-layer OLEDs [25]. In general, the doping of emission dyes tends to promote or restrain the current density. The promoting behavior means that the dopant molecules assist the transport of carriers. In contrast, the decrease of current density implies that the dopant molecules act as trapping sites for carriers [26]. To further elucidate the effect of dopant molecules on electrical property, single carrier devices H_1_–H_2_ and E_1_–E_2_ based on device B with the doping ratio CBP to TPBi of 2:1 are prepared. The hole-only devices H_1_–H_2_ include following layers: ITO/MoO_3_ (2 nm)/NPB (40 nm)/CBP: TPBi: PO-01 (30 nm)/NPB (40 nm)/MoO_3_ (2 nm)/Al. The configuration of the electron-only devices: ITO/Liq (1 nm)/TPBi (40 nm)/CBP: TPBi: PO-01 (30 nm)/TPBi (40 nm)/Liq (1 nm)/Al. The concentration of PO-01 dopant denotes 0% for devices H_1_ and E_1_, 15% for devices H_2_ and E_2_.

**Figure 4:**
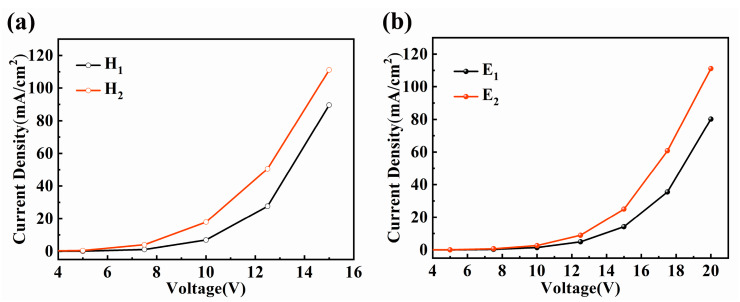
Current density–voltage curves of (**a**) hole-only devices H_1_–H_2_ and (**b**) electron-only devices E_1_–E_2_.

It is shown that the hole-only current density in Figure 4a is indeed greatly increased when doping 15% PO-01 into EML. Clearly, since the highest occupied molecular orbital (HOMO) of PO-01 (5.1 V) is significantly shallower than that of CBP (6.0 V) and TPBi (6.3 V), the lower energy barrier between the MoO_3_ and PO-01 facilitates the injection of holes. The increasing behavior suggests that PO-01 molecules create an additional hole-transporting channel through hole hopping transport in the doping structure of CBP:TPBi:15% PO-01, which promotes the transport of holes and hence the charge balance in the EML [27]. As shown in Figure 4b, doping PO-01 into EML depicts higher current density compared with the undoped device. The obvious enhancement should arise from the fact that PO-01 molecules form an effectively continuous electron-transporting channel as well, which is favorable to promote the transport of electrons and avoid spectral instability due to the electron-trapping behavior in the single-layer devices [28]. In summary, it is worth noting that the PO-01 dopant tends to act as additional transporting channels of both holes and electrons simultaneously. Therefore, the transport property of charge carriers is increased upon doping PO-01 into the co-host system, resulting in increased exciton recombination efficiency, which is greatly beneficial to high efficiency single-layer OLEDs.

## 4. Conclusions

In conclusion, the superior efficiency of orange phosphorescent single-layer OLEDs with excellent charge transport properties has been demonstrated by using a uniform co-host configuration doped with PO-01 as the EML. The single-layer device possesses maximum CE and PE of 37.1 cd A^−1^ and 33.3 lm W^−1^, which are obtained by optimizing the composition ratio of the co-host materials. Also, the electrical property and the exciton recombination region and of PO-01-doped devices are also discussed in detail. It is found that the enhancement of charge transport inspired by PO-01 and suitable location of the exciton recombination region make for a more balanced charge carrier transport. The remarkable performance should be attributed to the utilization of CBP:TPBi bipolar host in EML and the dopant effect on the promotion of carrier injection and transport.

## Figures and Tables

**Table 1:** Detailed performance of devices A–D. CIE(x,y): defined as CIE coordinate at 1000 cd m^−2^; V_on_: defined as the drive voltage at 1 cd m^−2^; Ƞ_max_/Ƞ_500_/Ƞ_1000_/Ƞ_3000_: maximum efficiency, efficiencies at 500, 1000 and 3000 cd m^−2^.

Devices	CIE(x,y)	V_on_	CE (cd A^−1^)	PE (lm W^−1^)
			Ƞ_max_/Ƞ_500_/Ƞ_1000_/Ƞ_3000_	Ƞ_max_
Device A	(0.51,0.48)	3.42	34.2/16.5/16.0/14.5	30.7
Device B	(0.51,0.48)	3.31	37.1/23.5/22.1/20.0	33.3
Device C	(0.51,0.48)	3.91	33.3/16.2/15.7/14.3	24.6
Device D	(0.51,0.48)	4.81	20.7/20.5/19.5/18.7	6.9
